# Metformin Suppresses Metastatic Potential and Sensitizes Ovarian Cancer Cells to Chemotherapeutics

**DOI:** 10.3390/ijms27177779

**Published:** 2026-08-30

**Authors:** Megha J. Pandya, Ayokunnumi Ogunsanya, Adedoyin Ajibade, Achuth Padmanabhan

**Affiliations:** 1 Department of Biological Sciences, University of Maryland Baltimore County, 1000 Hilltop Circle, Baltimore, MD 21250, USA; meghajp1@umbc.edu (M.J.P.); aogunsa2@umbc.edu (A.O.); aajibad1@umbc.edu (A.A.); 2 University of Maryland Greenbaum Comprehensive Cancer Center, Baltimore, MD 21201, USA

**Keywords:** ovarian cancer, metastatic potential, metformin, therapeutic combination, carboplatin, drug repurposing, FDA-approved drugs

## Abstract

The failure of current therapeutics to elicit a durable response in metastatic ovarian cancer patients highlights the urgent need to develop more effective treatment strategies. In this study, we demonstrate that the anti-diabetic drug metformin exerts cytotoxic effects across a broad panel of ovarian cancer cell lines. Transcriptomic analyses revealed that, in addition to reducing cell viability and proliferation, metformin modulates pathways associated with metastatic progression, including cell migration, extracellular matrix remodeling, and cellular responses to chemotherapeutic agents. Consistent with these molecular changes, metformin significantly impaired ovarian cancer cell migration and invasion through Matrigel. Furthermore, metformin diminished the ability of ovarian cancer cells to form multicellular aggregates, a key property that facilitates successful metastatic dissemination within the peritoneal cavity. Although metformin produced only a modest increase in carboplatin sensitivity, systematic screening of FDA-approved drugs identified several agents whose efficacy was enhanced by metformin, including drugs not currently used to treat ovarian cancer. Collectively, these findings demonstrate that metformin suppresses metastatic phenotypes and enhances therapeutic vulnerability in ovarian cancer cells, supporting its potential repurposing as a combination therapy to improve treatment outcomes in ovarian cancer.

## 1. Introduction

Ovarian cancer is an extremely lethal gynecologic malignancy with limited therapeutic options [1]. Due to the absence of reliable early diagnostic markers, the majority of patients continue to be diagnosed with advanced metastatic disease [1]. First-line treatment for these patients includes cytoreductive surgery followed by chemotherapy using a combination of platinum and taxane-based compounds [2]. Although most patients respond well to this strategy initially, in over 85% of patients, the tumor relapses within 6 months [3]. Relapsed tumors are resistant to extant therapeutics, resulting in poor clinical outcomes [1,4,5]. The failure of extant therapeutics to provide durable responses highlights the urgent need to identify novel therapeutic strategies and drug combinations that will help overcome chemoresistance and improve clinical outcomes in ovarian cancer patients.

Drug repositioning or repurposing is the process of using an existing drug for the treatment of a medical condition for which it has not been previously used [6]. As these drugs have already undergone rigorous safety evaluation, their transition into the clinic can be achieved much more efficiently and at lower costs as compared to conventional drug development routes. Metformin is an FDA-approved first-line therapeutic for type 2 diabetes [7,8,9]. Metformin has been in clinical use for over 70 years and has a well-documented safety profile [7,8,9,10]. More recently, metformin has attracted considerable attention for its potential as an anticancer agent. Several epidemiological studies have reported a reduced incidence of cancer among patients with diabetes who are treated with metformin [11,12,13]. Metformin exerts both direct and indirect effects on cancer cells. Its direct effects include activation of AMP-activated protein kinase (AMPK), which, among other downstream effects, inhibits mammalian target of rapamycin (mTOR) signaling [14]. Indirectly, metformin enhances insulin sensitivity and suppresses hepatic gluconeogenesis, thereby reducing circulating insulin levels and attenuating insulin-mediated phosphatidylinositol 3-kinase (PI3K) signaling [14]. Metformin has also been reported to suppress ERK and STAT3 signaling, key pathways involved in the regulation of cellular proliferation and apoptosis [14]. Combining metformin with other chemotherapeutics has been shown to be an effective strategy in several different cancers. For instance, metformin was shown to enhance the anti-cancer effects of cytarabine in acute myeloid leukemia (AML), an effect that was attributed to the inhibition of the mTORC1/p70S6K pathway [15]. Studies using MCF10A ER-Src breast cancer xenografts in nude mice showed that metformin exerts selective cytotoxicity against cancer stem cells [16]. Further, metformin synergized with doxorubicin to inhibit breast tumor growth in mice [16]. Additionally, metformin has been shown to enhance the sensitivity of sarcomas and non-small cell lung cancer to radiation in an AMPK-dependent manner [17].

A recent study reported that metformin use was associated with improved survival in patients with ovarian cancer [18]. However, because these findings were derived primarily from cohorts of patients with diabetes, their generalizability to non-diabetic individuals remains uncertain. Moreover, diabetes itself may influence cancer progression and treatment response, potentially confounding the observed effects of metformin. Therefore, prospective studies evaluating metformin in non-diabetic patients with ovarian cancer will be important to better define its therapeutic potential. Meta-analysis suggested that metformin may have benefits as an adjuvant agent for gynecological cancer in patients with type-2 diabetes [19]. Early phase clinical studies further demonstrated that metformin is well tolerated and may reduce ovarian cancer stem cell populations while enhancing platinum sensitivity [20]. However, the first randomized placebo-controlled Phase II trial found that the addition of metformin to standard first-line chemotherapy did not improve progression-free or overall survival [21]. These findings underscore the need to identify novel therapeutic combinations in which metformin may provide greater clinical benefit, particularly combinations involving agents not previously established as therapeutic strategies for ovarian cancer. The present study seeks to address this knowledge gap by investigating whether metformin can enhance the efficacy of such agents and thereby uncover new therapeutic opportunities for ovarian cancer.

In this study, we provide evidence that metformin exerts potent anti-cancer effects across a broad spectrum of ovarian cancer cells and can enhance their sensitivity to pharmacologically distinct agents. Using a panel of FDA-approved small molecules, we identified several compounds whose cytotoxic effects were markedly enhanced in the presence of metformin, including agents not previously established as therapeutic strategies for ovarian cancer. These findings suggest that metformin may have therapeutic utility beyond its intrinsic anti-cancer activity by broadening the efficacy of existing drugs and enabling the therapeutic repurposing of agents with previously unrecognized potential in ovarian cancer. Collectively, our study identifies novel drug combinations that warrant further investigation as potential therapeutic strategies for ovarian cancer.

## 2. Results and Discussion

### 2.1. Metformin Decreases Cell Viability and Proliferation in Ovarian Cancer Cells

**Figure 1:**
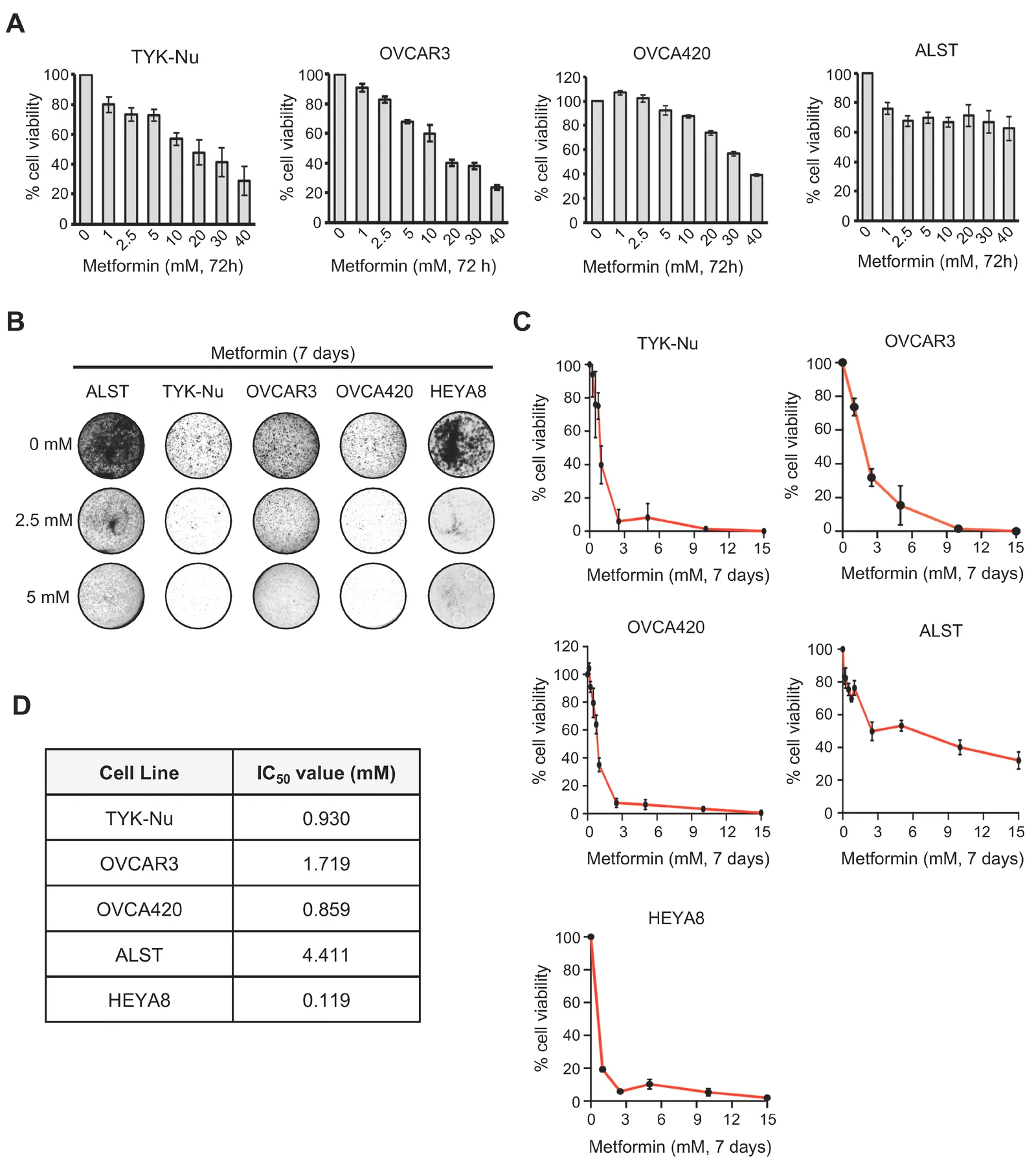
Metformin decreases cell viability and proliferation in ovarian cancer cells. (**A**) WST-1 cell viability assay showing the sensitivity of ovarian cancer cells to short-term metformin treatment (*n* = 3, 72 h). (**B**) Representative crystal violet staining showing the effect of long-term metformin treatment on ovarian cancer cells. (**C**) Quantified data from crystal violet staining shown in Figure 1B showing the effect of long-term metformin treatment on ovarian cancer cells (*n* = 3; mean + standard deviation). (**D**) Calculated IC_50_ values for metformin across different ovarian cancer cell lines based on the dose–response data presented in Figure 1C.

To investigate the therapeutic potential of metformin in ovarian cancer, we treated a panel of ovarian cancer cell lines with increasing concentrations of metformin for varying durations. A WST-1 cell viability assay performed after 72 h of treatment revealed a dose-dependent reduction in cell viability in TYK-Nu, OVCAR3, and OVCA420 cells, but not in ALST cells (Figure 1A). To assess the longer-term effects of metformin on ovarian cancer cells, we performed crystal violet assays following metformin treatment of TYK-Nu, OVCAR3, OVCA420, ALST, and HEYA8 cells. TYK-Nu, OVCAR3, and OVCA420 cells were assessed 7 days after treatment, whereas ALST and HEYA8 cells were assessed 5 days after treatment. Long-term metformin treatment resulted in a pronounced, dose-dependent reduction in cell viability across all ovarian cancer cell lines tested (Figure 1B–D). Importantly, metformin had minimal effect on the viability of the non-cancerous immortalized fallopian tube epithelial cell line FT237 (Figure S1A,B). Collectively, these findings demonstrate that metformin exerts potent dose- and time-dependent effects on ovarian cancer cell viability and proliferation across a broad spectrum of ovarian cancer cell lines.

### 2.2. Metformin Alters Key Cellular Processes Underlying Ovarian Cancer Metastasis

**Figure 2:**
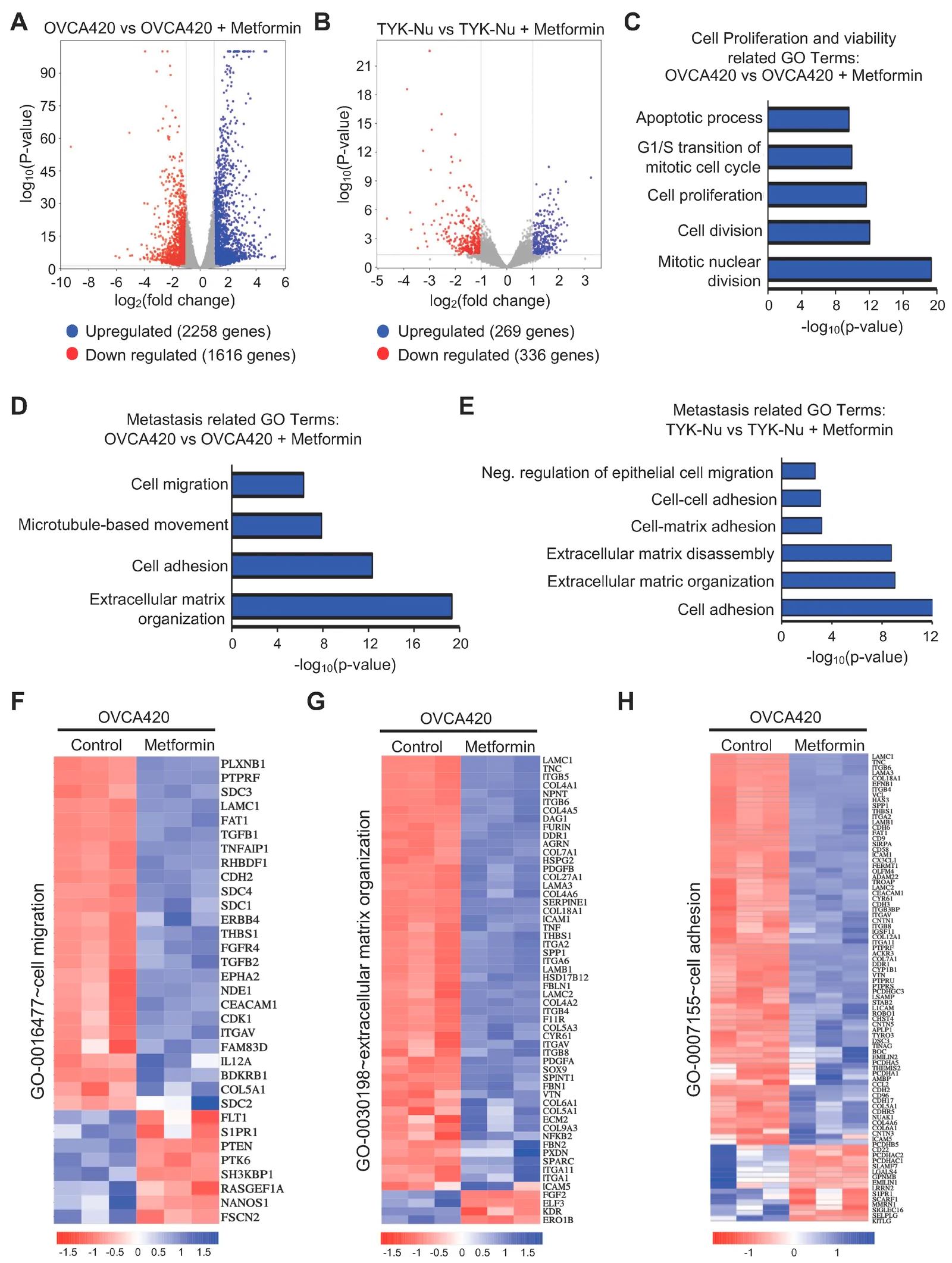
Metformin alters key cellular processes underlying ovarian cancer metastasis. (**A**) Volcano plot showing differentially regulated genes in metformin-treated OVCA420 cells (*n* = 3; *p*-value < 0.05; log2 fold change > 1). (**B**) Volcano plot showing differentially regulated genes in metformin-treated TYK-Nu cells (*n* = 3; *p*-value < 0.05; log2 fold change > 1). (**C**) Gene ontology terms that are significantly enriched in the differentially expressed gene sets between control and metformin-treated OVCA420 cells that are related to cell viability and proliferation. (**D**) Gene ontology terms that are significantly enriched in the differentially expressed metastasis-related gene sets between control and metformin-treated OVCA420 cells. (**E**) Gene ontology terms that are significantly enriched in the differentially expressed metastasis-related gene sets between control and metformin-treated TYK-Nu cells. (**F**) Heatmap showing differentially regulated genes in metformin-treated ovarian cancer cells related to cell migration. (**G**) Heatmap showing differentially regulated genes in metformin-treated OVCA420 cells related to extracellular matrix reorganization. (**H**) Heatmap showing differentially regulated genes in metformin-treated OVCA420 cells related to cell adhesion.

To understand how metformin impacts molecular processes in ovarian cancer cells, we performed RNA sequencing using control and metformin-treated OVCA420 and TYK-Nu cells. Metformin treatment resulted in a total of 3874 genes in OVCA420 cells (Figure 2A and Table S1) and 605 genes in TYK-Nu cells (Figure 2B and Table S2) being differentially regulated. Among these, 2258 genes were upregulated and 1616 genes were downregulated in OVCA420 cells (Figure 2A). In comparison, 269 genes were upregulated and 336 genes were downregulated in TYK-Nu cells (Figure 2B). To better understand how such a massive alteration in gene expression impacts biological processes, we performed Gene Ontology (GO) Analysis using the differentially regulated genes. GO Analysis revealed that several processes important in cell proliferation and viability were altered upon metformin treatment (Figure 2C, Tables S3 and S4). These molecular alterations are consistent with the impact metformin has on ovarian cancer proliferation and viability. In addition to these, GO Analysis also revealed that metformin treatment in both OVCA420 and TYK-Nu cells resulted in molecular changes associated with metastatic phenotypes such as cell migration, extracellular matrix reorganization, cell adhesion, and cell–matrix adhesion (Figure 2D–H and Figure S2A,B). These data suggested that in addition to its effect on cell viability and proliferation, metformin reprograms key signaling networks that impact the metastatic phenotype in ovarian cancer cells. We focused on investigating metastatic phenotypes further because approximately 85% of ovarian cancer patients are diagnosed at an advanced stage, when the disease has already disseminated beyond the primary tumor. Thus, an effective therapeutic strategy should ideally not only inhibit tumor cell proliferation and viability but also target metastatic dissemination.

### 2.3. Metformin Impairs Metastatic Phenotypes in Ovarian Cancer Cells

**Figure 3:**
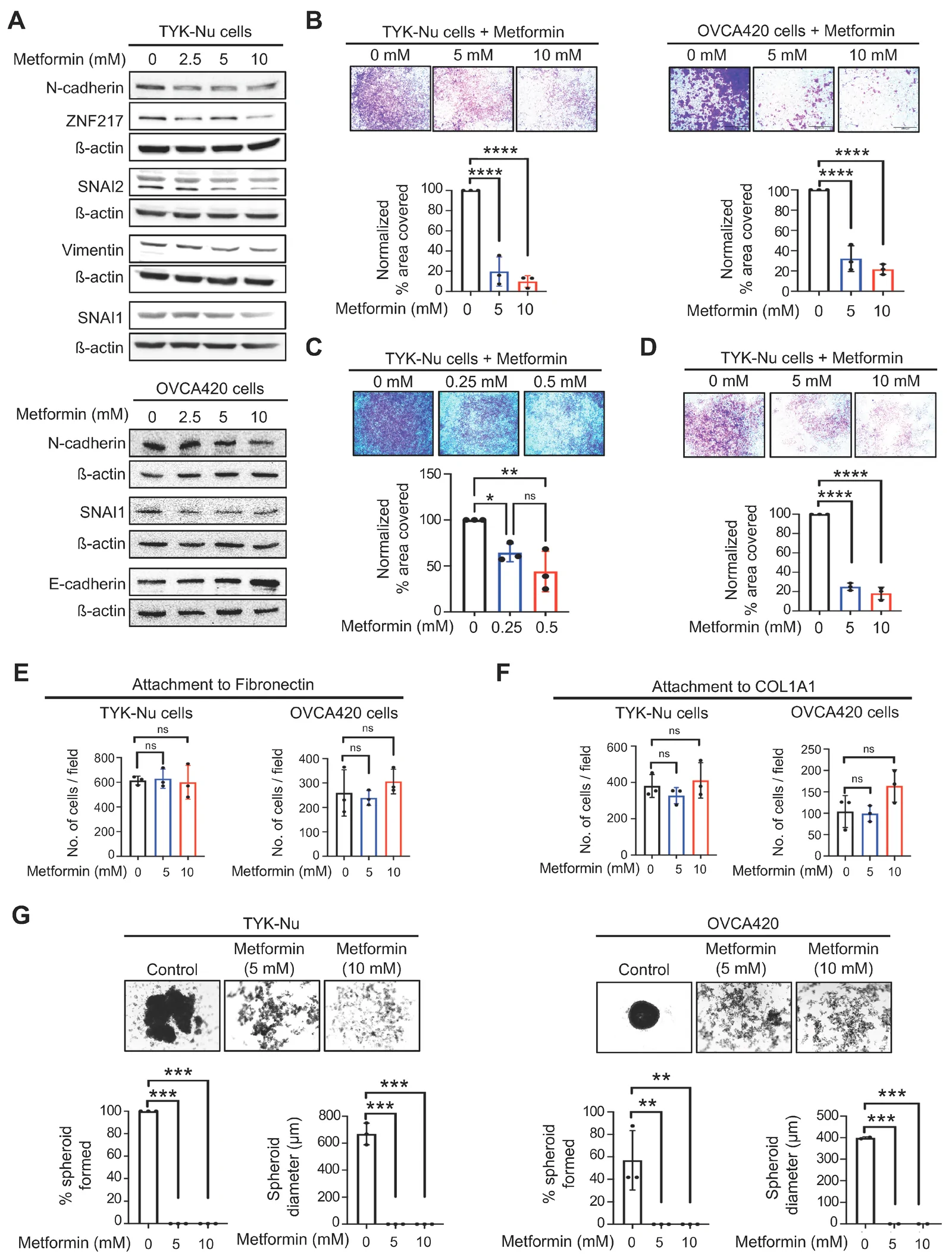
Metformin impairs metastatic potential of ovarian cancer cells. (**A**) Western blot reveals alteration in mesenchymal and epithelial markers in TYK-Nu cells upon metformin treatment. (**B**) Metformin decreases cell migration in TYK-Nu and OVCA420 cells (transwell migration assay). (**C**) Metformin impairs cell migration in TYK-Nu cells even at lower concentrations. (**D**) Metformin reduces invasive potential of TYK-Nu cells (Matrigel invasion assay). Metformin does not impact the attachment of ovarian cancer cells to (**E**) fibronectin and (**F**) COL1A1. (**G**) Metformin reduces the number and size of spheroids formed by ovarian cancer cells. *p* ≤ 0.05 (*), *p* ≤ 0.01 (**), *p* ≤ 0.001 (***), and *p* ≤ 0.0001 (****).

Enhanced ability to migrate and invade through the extracellular matrix is critical for cancer cells to successfully metastasize from the primary tumor site to distant organs. Our RNA-seq data suggest that metformin treatment alters gene expression programs associated with these metastatic traits in ovarian cancer cells (Figure 2). As changes in a cell’s migratory and invasive potential are often associated with changes in the cellular state from epithelial to mesenchymal or vice versa, we evaluated the effect of metformin on established epithelial–mesenchymal transition (EMT) markers. Consistent with the observed transcriptomic changes, metformin treatment resulted in a dose-dependent decrease in mesenchymal markers such as N-cadherin, ZNF217, SNAI1, SNAI2, and vimentin (Figure 3A). These results suggest a shift from a mesenchymal to a more epithelial state in these cells. As the shift in cellular state from a mesenchymal to an epithelial phenotype can impair the ability of cancer cells to migrate and invade through the extracellular matrix, we next investigated whether metformin alters these metastatic properties. To determine the impact of metformin on cellular motility, we assessed the migratory capacity of ovarian cancer cells using a transwell migration assay. Metformin significantly reduced the ability of TYK-Nu and OVCA420 cells to migrate (Figure 3B). Because it is important to distinguish the anti-metastatic effects of metformin from its cytotoxic effects, we evaluated the effect of metformin on cell viability in TYK-Nu and OVCA420 cells over the 24 h period corresponding to the duration of our migration and invasion assays. Metformin treatment for 24 h did not significantly affect cell viability in either OVCA420 or TYK-Nu cells, indicating that the inhibitory effects of metformin on cell migration and invasion are unlikely to be attributable to reduced cell viability during the assay period (Figure S3A). Additionally, metformin was effective in impairing migration in ovarian cancer cells even at much lower concentrations, providing additional evidence that the effects of metformin on metastatic phenotypes are not simply a consequence of generalized cytotoxicity (Figure 3C and Figure S3B). The capacity to invade through the extracellular matrix is a hallmark of metastatic ovarian cancer cells and is required for efficient metastatic seeding and progression. To determine the impact of metformin on the invasive potential of ovarian cancer cells, we performed a Matrigel invasion assay. In comparison to vehicle-treated cells, metformin-treated TYK-Nu cells exhibited a significant reduction in invasive potential (Figure 3D). These results suggest that metformin can negatively impact the ability of ovarian cancer cells to migrate and invade through the extracellular matrix.

Attachment to extracellular matrix proteins provides traction needed for cell movement through surrounding tissues and is essential for metastatic tumor cell migration. To test how metformin impacted the ability of ovarian cancer cells to attach to extracellular matrix proteins, we assessed the attachment of control and metformin-treated TYK-Nu and OVC420 cells to fibronectin and COLA1-treated plates. Metformin treatment did not have any impact on the ability of these cells to attach to these ECM proteins (Figure 3E,F). During ovarian cancer metastasis, cancer cells are shed from the primary tumor into the peritoneal space [22]. The ability of these cells to form multicellular aggregates or spheroids is a critical step in the metastatic progression. Metformin treatment completely blocked the ability of TYK-Nu and OVCA420 cells to form multicellular spheroids when grown in low attachment plates (Figure 3G). This result suggests that metformin can impair their ability to form multicellular aggregates. Thus, our data show that metformin decreases the metastatic potential of ovarian cancer cells by impairing several processes that are critical for successful metastasis.

### 2.4. Metformin Sensitizes Ovarian Cancer Cells to Chemotherapeutic Drugs

**Figure 4:**
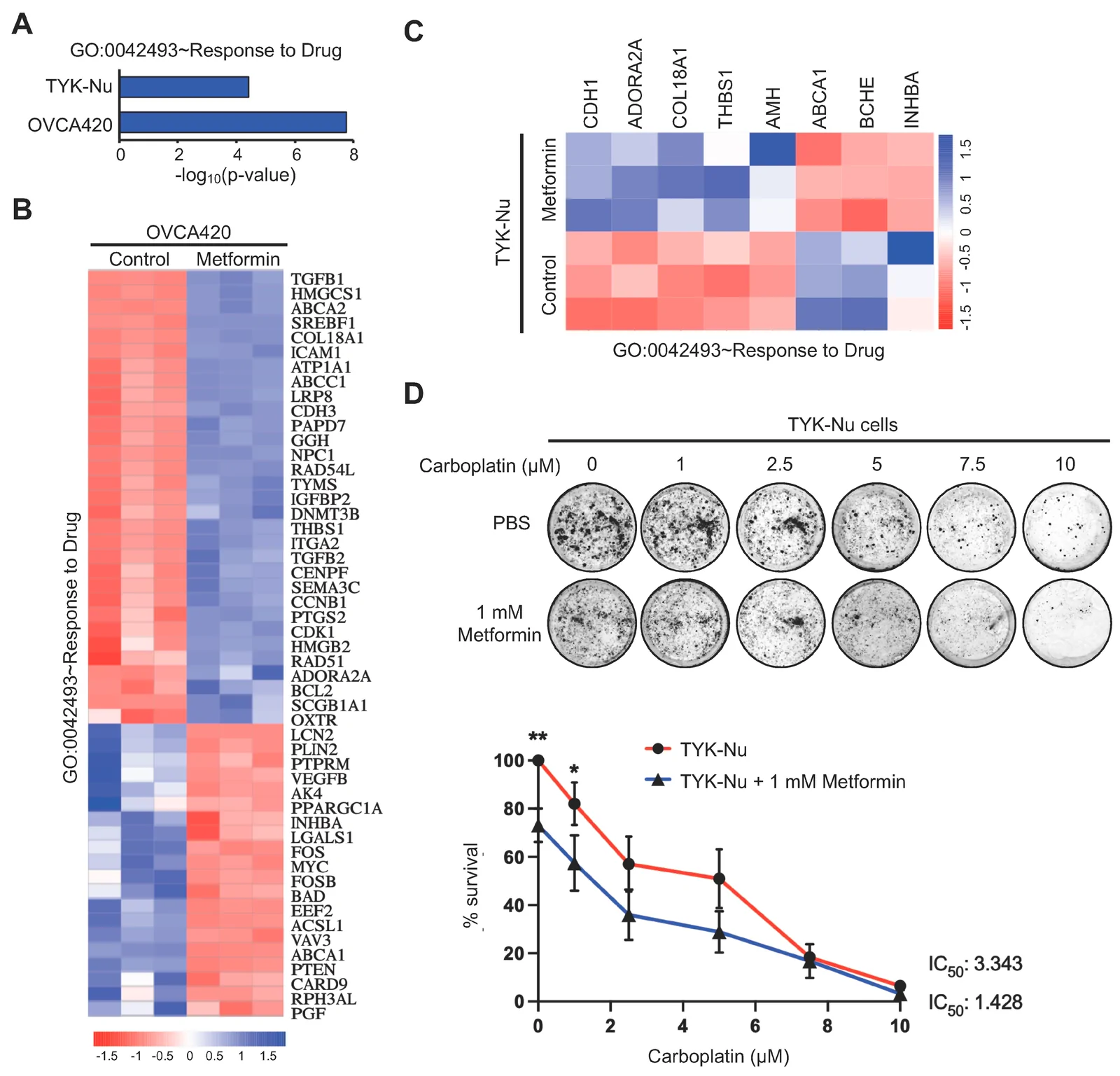
Metformin impacts chemoresistance in ovarian cancer cells. (**A**) Gene Ontology Analysis revealed that drug response-related biological processes were significantly enriched among the differentially expressed genes in metformin-treated OVCA420 and TYK-Nu cells compared with control cells. (**B**) Heatmap showing differentially regulated genes in metformin-treated OVCA420 cells related to drug response. (**C**) Heatmap showing differentially regulated genes in metformin-treated TYK-Nu cells related to drug response. (**D**) Cell viability data show that metformin enhances carboplatin’s cytotoxicity in TYK-Nu cells. *p* ≤ 0.05 (*), *p* ≤ 0.01 (**).

Chemoresistance is a major contributor to poor clinical outcomes in patients with metastatic ovarian cancer [23]. Analysis of RNA-sequencing data from TYK-Nu and OVCA20 cells revealed that metformin treatment significantly altered molecular pathways associated with drug response (Figure 4A). Consistent with this finding, numerous genes involved in drug sensitivity and resistance were differentially expressed in both OVCA420 and TYK-Nu cells following metformin treatment (Figure 4B,C). Given that carboplatin is a cornerstone of frontline therapy for ovarian cancer and that acquired resistance to carboplatin remains a major clinical challenge, we next examined whether metformin could enhance the cytotoxic effects of carboplatin in ovarian cancer cells. To this end, we evaluated the effects of metformin in combination with carboplatin following long-term treatment (7 days) in TYK-Nu cells. The combination of metformin and carboplatin resulted in greater overall cytotoxicity than either treatment alone, indicating that the combination may enhance the antitumor effects of carboplatin (Figure 4D).

**Figure 5:**
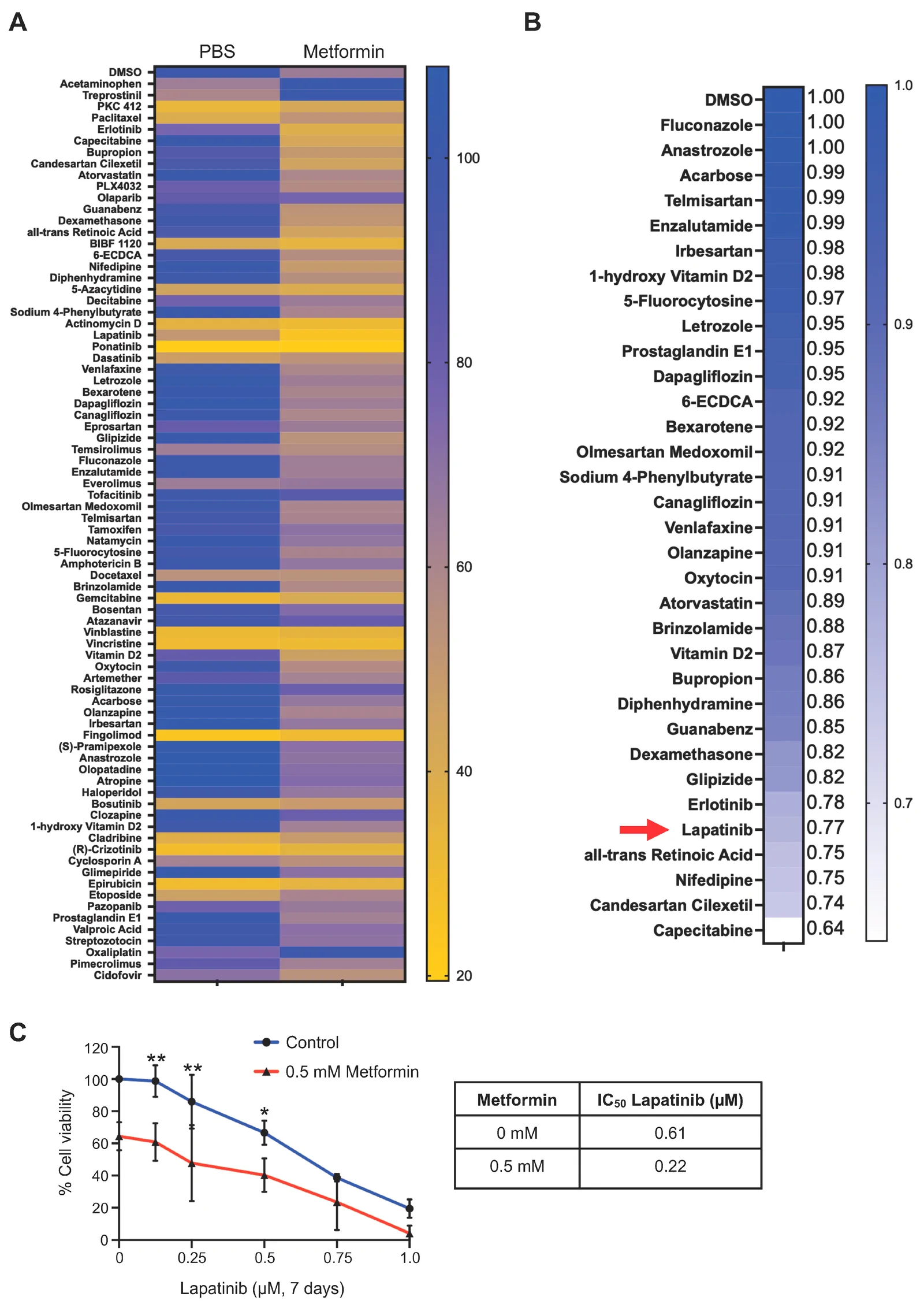
Metformin sensitizes ovarian cancer cells to chemotherapeutic drugs. (**A**) Heatmap from WST-1 cell viability data in TYK-Nu cells treated with 10 µM of the different FDA-approved small molecules either by itself or in combination with 5 mM metformin for 48 h. (**B**) Heatmap showing small molecules whose cytotoxicity in TYK-Nu cells was enhanced by metformin. The values are represented as normalized fold changes to the cytotoxic effect of the drug alone. Lapatinib is indicated using a red arrow. (**C**) Metformin (0.5 mM) enhances the cytotoxicity of lapatinib in TYK-Nu cells. *p* ≤ 0.05 (*), *p* ≤ 0.01 (**).

To determine if metformin can enhance the efficacy of other clinically translatable chemotherapeutic agents, we screened a panel of FDA-approved drugs in TYK-Nu cells treated with either vehicle (PBS) or metformin. The use of a diverse panel of clinically approved compounds with distinct mechanisms of action provides an opportunity to broadly assess how metformin modulates the therapeutic response of ovarian cancer cells to agents targeting different cellular pathways. This approach may facilitate the identification of previously unrecognized drug combinations and provide insights into the broader mechanisms through which metformin enhances drug sensitivity. Cell viability was assessed after 48 h of drug treatment. This time point was selected because the relatively short treatment duration facilitates identification of compounds that produce the most potent cytotoxic responses when combined with metformin. We found that co-treatment with metformin enhanced the cytotoxicity of 34 FDA-approved compounds (Figure 5A,B, and Table 1). To calculate normalized cytotoxicity presented in Figure 5B and Table 1, we first determined the fold change in cell viability for each drug in the presence of metformin relative to the corresponding drug treatment alone. These values were then normalized to the fold change observed with metformin treatment alone. Importantly, metformin alone reduced cell viability by approximately 40% (i.e., to 0.60 relative to untreated control). Thus, a further reduction of 36% viability (0.64 relative to metformin-treated cells) with the combination of capecitabine corresponds to an overall viability of approximately 0.24 relative to untreated control, representing a 76% reduction in cell viability. Notably, several of these agents have not previously been investigated as therapeutics for ovarian cancer. The top six hits identified in our screen were capecitabine, candesartan cilexetil, Nifedipine, all-trans retinoic acid, lapatinib, and erlotinib. Future studies should focus on characterizing the effect of combining metformin with these drugs in clinically relevant in vitro as well as in vivo models of ovarian cancer. Overall, these findings support further investigation of metformin in combination with existing therapeutics and with agents identified through our screening approach.

## 3. Materials and Methods

### 3.1. Chemicals and Reagents

RPMI-1640 media containing L-glutamine was purchased from Corning (#10-040-CMR, Corning, NY, USA). Fetal Bovine Serum (FBS) was obtained from Omega Scientific (#FB-02, Tarzana, CA, USA). Pen-Strep was purchased from Gibco (#15140163, Grand Island, NY, USA). WST-1 reagent was purchased from Millipore-Sigma (#11644807001, Burlington, MA, USA). PBS was obtained from Corning (#21-040-CV) and 4% paraformaldehyde was purchased from Thermofisher (#J19943, Waltham, MA, USA). An 8 µm, 24-well permeable insert was purchased from Greiner Bio-One Thincert™ (#662638, Kremsmünster, Austria). Matrigel was purchased from Corning (#354234, NY, USA). Fluromount-G mounting media was purchased from Thermofisher (#00-4958-02, Waltham, MA, USA). Metformin (#13118), Carboplatin (#13112) and FDA-approved drug library (#23538) were purchased from Cayman Chemical (Ann Arbor, MI, USA).

### 3.2. Cell Lines and Culture Conditions

OVCA420 and TYK-Nu cells were a kind gift from KK Wong (MD Anderson Cancer Center, Houston, TX, USA). FT237 immortalized fallopian tube cell lines were a kind gift from Dr. Ronny Drapkin (University of Pennsylvania, Philadelphia, PA, USA). All human ovarian cancer cell lines used in this study were grown in RPMI-1640 containing L-glutamine supplemented with 10% FBS and 1% Pen-Strep in a 37 °C incubator with 5% CO_2_. All cell lines were routinely tested for mycoplasma and used at low passage numbers.

### 3.3. Western Blot Analysis

Cells were lysed using RIPA buffer supplemented with protease and phosphatase inhibitors. Protein concentrations were determined using Bio-Rad Protein Assay Dye Reagent. An equal amount of total protein was run using an SDS-PAGE gel and transferred to a PVDF membrane. The membrane was blocked for 1 h at room temperature in 2% milk and incubated with primary antibodies overnight at 4 °C on a shaker. The membranes were washed three times with 1X TBST, incubated with appropriate HRP-linked secondary antibodies for 1 h at room temperature on a shaker, washed again with 1X TBST three times, developed using enhanced chemiluminescence reagent, and imaged using a BioRad ChemiDoc Imager (Hercules, CA, USA).

### 3.4. Cell Viability Assays

For short-term drug viability assays, 5000 cells were seeded in each well of a 96-well plate. After 24 h incubation at 37 °C with 5% CO_2_, the cells were treated with respective drugs and incubated for an additional 48 h. Viable cells in each well were determined using WST-1 reagent as per the manufacturer’s recommendation. For long-term cell viability assays, 5000 cells were seeded in each well of a 6-well plate. After 24 h incubation at 37 °C with 5% CO_2_, the cells were treated with different chemotherapeutic drugs. TYK-Nu, OVCA420 and OVCAR3 cells were incubated for an additional 7 days. ALST and HEYA8 cells were incubated for 5 days. Post-drug treatment, the cells were washed with 1x PBS, fixed with 4% paraformaldehyde and stained using 0.5% crystal violet. Additional crystal violet was removed by washing the wells with distilled water. Cells were imaged using a microscope (AmScope, Irvine, CA, USA) and quantified with ImageJ (version 1.54t).

### 3.5. Transwell Cell Migration Assays

An amount of 40,000 cells (TYK-Nu) and 80,000 (OVCA420) were resuspended in RPMI-1640 media containing 10% FBS and seeded inside 8 µM 24-well transwell inserts. Inserts were placed in a 24-well plate containing RPMI-1640 media with 20% FBS and incubated at 37 °C with 5% CO_2_ for 24 h and 42 h, respectively. Subsequently, the inserts were washed with 1X PBS, fixed with 4% paraformaldehyde, stained using 0.5% crystal violet, imaged using a microscope and quantified with ImageJ.

### 3.6. Matrigel Cell Invasion Assays

Corning Matrigel^®^ Matrix was diluted at a ratio of 1:3 with serum-free RPMI-1640 containing L-glutamine. A 24-well permeable 8 µm insert was coated with 30 µL of diluted Matrigel and allowed to jellify for 30 min at room temperature. An amount of 40,000 TYK-Nu cells in media lacking serum was seeded inside the Matrigel-coated inserts. Inserts were placed in a well containing media with 20% Fetal Bovine serum. Cells were incubated for 48 h and 66 h for TYK-Nu and OVCA420, respectively, washed with 1X PBS, fixed with 4% paraformaldehyde, stained with 0.5% crystal violet, and quantified using ImageJ.

### 3.7. Spheroid Formation Assay

For the spheroid formation assay, 2500 cells were resuspended in 10 µL of culture medium and used to generate hanging drops as previously described (PMID: 21587162). Metformin was added at the indicated concentrations at the time of hanging-drop setup. Plates were incubated at 37 °C in a humidified CO_2_ incubator for 48 h to allow spheroid formation. Spheroids were then gently transferred to non-adherent plates containing the appropriate culture medium and incubated for an additional 24 h under the same conditions. Spheroids were imaged using a microscope at 4× magnification, and spheroid diameter was quantified using ImageJ.

### 3.8. RNA Sequencing and Analysis

Control and metformin-treated cell pellets were shipped to Azenta Life Sciences (South Plainfield, NJ, USA). Total RNA was extracted from fresh frozen tissue samples using Qiagen RNeasy Plus Universal mini kit following manufacturer’s instructions (Qiagen, Hilden, Germany). RNA samples were quantified using a Qubit 4 Fluorometer (Life Technologies, Carlsbad, CA, USA) and RNA integrity was checked using Agilent TapeStation 4200 (Agilent Technologies, Palo Alto, CA, USA). ERCC RNA Spike-In Mix kit (cat. 4456740) from ThermoFisher Scientific was added to normalized total RNA prior to library preparation following the manufacturer’s protocol. RNA sequencing libraries were prepared using the NEBNext Ultra II RNA Library Prep Kit for Illumina using manufacturer’s instructions (NEB, Ipswich, MA, USA). Briefly, mRNAs were initially enriched with Oligod(T) beads. Enriched mRNAs were fragmented for 15 min at 94 °C. First-strand and second-strand cDNA were subsequently synthesized. cDNA fragments were end-repaired and adenylated at 3′ ends, and universal adapters were ligated to cDNA fragments, followed by index addition and library enrichment by PCR with limited cycles. The sequencing library was validated on the Agilent TapeStation (Agilent Technologies, Palo Alto, CA, USA), and quantified by using a Qubit 4 Fluorometer (Invitrogen, Carlsbad, CA, USA) as well as by quantitative PCR (KAPA Biosystems, Wilmington, MA, USA). The sequencing libraries were clustered on 1 lane of a flowcell. After clustering, the flowcell was loaded on the Illumina instrument (HiSeq 4000 or equivalent) according to manufacturer’s instructions. The samples were sequenced using a 2 × 150 bp Paired End (PE) configuration. Image analysis and base calling were conducted by the Control software. Raw sequence data (.bcl files) generated by the sequencer were converted into fastq files and de-multiplexed using Illumina’s bcl2fastq 2.17 software. One mismatch was allowed for index sequence identification. After investigating the quality of the raw data, sequence reads were trimmed to remove possible adapter sequences and nucleotides with poor quality. The trimmed reads were mapped to the GRCm38 reference genome available on ENSEMBL using the STAR aligner v.2.5.2b. The STAR aligner is a splice aligner that detects splice junctions and incorporates them to help align the entire read sequences. BAM files were generated as a result of this step. Unique gene hit counts were calculated by using featureCounts from the Subread package v.1.5.2. Only unique reads that fell within exon regions were counted. After extraction of gene hit counts, the gene hit counts table was used for downstream differential expression analysis. Using DESeq2, a comparison of gene expression between the groups of samples was performed. The Wald test was used to generate *p*-values and Log2 fold changes. Genes with adjusted *p*-values < 0.05 and absolute log2 fold changes >1 were called differentially expressed genes for each comparison. A gene ontology analysis was performed on the statistically significant set of genes by implementing the software GeneSCF. The mgi GO list was used to cluster the set of genes based on their biological process and determine their statistical significance. A Volcano plot and heatmap were generated using the SRPlot platform [24].

### 3.9. Quantification and Data Analysis

All in vitro assays were done in triplicate with multiple technical replicates each time. All data points on the graph were represented as the mean with standard deviation shown as error bars. Surface area covered by cells for in vitro assays was calculated using Image J/Fiji per field of image and normalized to the respective values in the control cells. Statistical analysis was done using a one-way ANOVA test with *p* ≤ 0.05 (*), *p* ≤ 0.01 (**), *p* ≤ 0.001 (***), and *p* ≤ 0.0001 (****). RNA-seq was performed in biological triplicate. Distribution of read counts in libraries was examined before and after normalization. The original read counts were normalized to adjust for various factors such as variations in sequencing yield between samples. These normalized read counts were used to accurately determine differentially expressed genes. The overall similarity among samples was assessed by the Euclidean distance between samples. This method was used to examine which samples are similar/different to each other and if they fit the expectation from the experiment design. A comparison of gene expression between the groups of samples was performed using DESeq2. The Wald test was used to generate *p*-values and log2 fold changes. Genes with an adjusted *p*-value < 0.05 and absolute log2 fold change >1 were called differentially expressed genes. Significantly differentially expressed genes were clustered by their gene ontology and the enrichment of gene ontology terms was tested using the Fisher exact test (GeneSCF v1.1-p2).

## 4. Conclusions

Metformin is a clinically approved drug that has been widely used for the treatment of type 2 diabetes for several decades [25,26]. Increasing evidence suggests that metformin also possesses anti-tumor activity across multiple cancer types, making it an attractive candidate for drug repurposing in oncology [27,28]. Our findings further support this concept by demonstrating that metformin simultaneously suppresses metastatic traits and enhances the sensitivity of ovarian cancer cells to multiple FDA-approved therapeutic agents. In addition, our study identifies several promising metformin-based combination therapies that warrant further preclinical and clinical evaluation. Given its well-established safety profile, low cost, and widespread clinical use, repurposing metformin in combination with existing therapeutics represents a promising approach that warrants further investigation for improving therapeutic responses in ovarian cancer.

Drug repurposing offers the advantage that the small molecules are already pre-selected for a favorable toxicity profile and pharmacokinetic properties. As these compounds are already in clinical use, their translation can skip many early preclinical and Phase I trials, which significantly accelerates timelines and reduces development costs. In addition to demonstrating the therapeutic benefit of combining metformin with carboplatin, the current frontline treatment for advanced ovarian cancer, we identified several other FDA-approved small molecules that have the potential to exhibit enhanced cytotoxicity in ovarian cancer cells when combined with metformin. Notably, many of these compounds have not previously been explored as therapeutic agents for ovarian cancer. Investigating the efficacy and underlying mechanisms of these novel drug combinations represents a promising direction for future studies and may facilitate the repurposing of clinically approved drugs for ovarian cancer treatment.

Many other reports have also suggested benefits of repurposing metformin as an ovarian cancer therapeutic [18,20]. Metformin’s ability to enhance the cytotoxicity of chemotherapeutic drugs has also been previously described in several cancers [27]. For instance, metformin was shown to synergize with the epidermal growth factor receptor inhibitor gefitinib and help overcome chemoresistance in non-small-cell lung cancer cells [29,30,31]. Metformin was also shown to sensitize cancer cells to sorafenib in non-small-cell lung cancer and hepatocellular carcinoma cells [32,33]. Importantly, co-treatment with metformin enables a 25% dose reduction in sorafenib without losing anti-tumor efficacy in thyroid cancer cells [34]. In breast cancer cells, combining metformin with the oral mTOR inhibitor everolimus resulted in a more potent anti-tumor effect [35]. In addition to these, metformin has also been shown to improve the efficacy of antibody-based drugs such as trastuzumab and bevacizumab [36,37]. These studies suggest that combining metformin with extant therapeutics is likely to yield better outcomes. Therefore, future studies should determine the ability of metformin to enhance the efficacy of extant drugs in gynecologic tumors such as ovarian cancer.

Although our study identifies several promising drug combinations, their therapeutic potential should be systematically evaluated across a diverse panel of ovarian cancer cell lines and validated in preclinical mouse models. While such studies are beyond the scope of the present work, comprehensive assessment of these combinations in both in vitro and in vivo models will be essential to establish their efficacy, identify the most promising candidates, and support future clinical translation. Taken together, our findings demonstrate that metformin exerts potent cytotoxic effects across a broad spectrum of ovarian cancer cells, suppresses their metastatic potential, and enhances the cytotoxic activity of multiple pharmacologically distinct agents. Importantly, our drug-screening approach identified several FDA-approved compounds, including agents not previously established as ovarian cancer therapeutics, whose activity was enhanced in the presence of metformin. These findings provide a rationale for further investigation of the most promising combinations through mechanistic studies to define the cellular pathways underlying their enhanced activity and to determine whether these interactions can be validated in relevant preclinical models. Future studies guided by these mechanistic insights may enable the rational prioritization of additional compounds and facilitate the development of metformin-based combination strategies with potential for clinical translation in ovarian cancer.

## Figures and Tables

**Table 1:** List of small molecules whose cytotoxicity in TYK-Nu cells was enhanced by metformin. The values are represented as normalized fold changes to the cytotoxic effect of the drug alone.

Name of the Drug	Normalized Cytotoxicity in the Presence of Metformin
DMSO (control)	1.00
Fluconazole	1.00
Anastrozole	1.00
Acarbose	0.99
Telmisartan	0.99
Enzalutamide	0.99
Irbesartan	0.98
1-Hydroxy Vitamin D2	0.98
5-Flourocytosine	0.97
Letrozole	0.95
Prostaglandin E1	0.95
Dapaglifozin	0.95
6-ECDCA	0.92
Bexarotene	0.92
Olmesartan Medoxomil	0.92
Sodium 4-Phenylbutyrate	0.91
Canagliflozin	0.91
Venlafaxine	0.91
Olanzapine	0.91
Oxytocin	0.91
Atorvastatin	0.89
Brinzolamide	0.88
Vitamin D2	0.87
Bupropion	0.86
Diphenhydramine	0.86
Guanabenz	0.85
Dexamethasone	0.82
Glipizide	0.82
Erlotinib	0.78
Lapatinib	0.77
All-Trans Retinoic Acid	0.75
Nifedipine	0.75
Candesartan Cilexetil	0.74
Capecitabine	0.64

## Data Availability

All data supporting the findings of this study are available within the article. RNA-seq data for control and metformin-treated TYK-Nu and OVCA420 cells used in this publication is available on the NCBI Sequence Read Archive (SRA) database (Accession Number—PRJNA1509624). There are no restrictions on data availability.

## Supplementary Materials

The following supporting information can be downloaded at: https://www.mdpi.com/article/10.3390/ijms27177779/s1.
