# Supplementary material for: Metformin Suppresses Metastatic Potential and Sensitizes Ovarian Cancer Cells to Chemotherapeutics
Source: Int J Mol Sci. 2026 Aug 30;27(17):7779. doi: 10.3390/ijms27177779 (PMC13566523; doi:10.3390/ijms27177779)
Supplement: Supplementary file 1 [file ijms-27-07779-s001.zip › Supplemental Data.pdf]

Supplemental Data

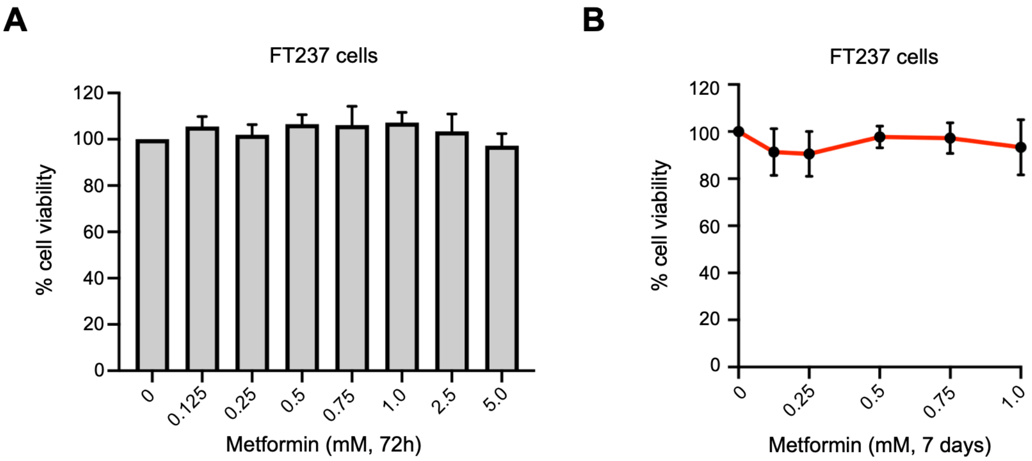

Figure S1. Metformin does not alter cell viability of immortalized fallopian tube cells, FT237. (A) WST-1 cell viability assay showing the effect of short-term metformin treatment (n=3, 72 h) on FT237 cells. (B) Quantified data from crystal violet staining showing the effect of long-term (7 days) metformin treatment on FT237 cells (n=3; mean + standard deviation).

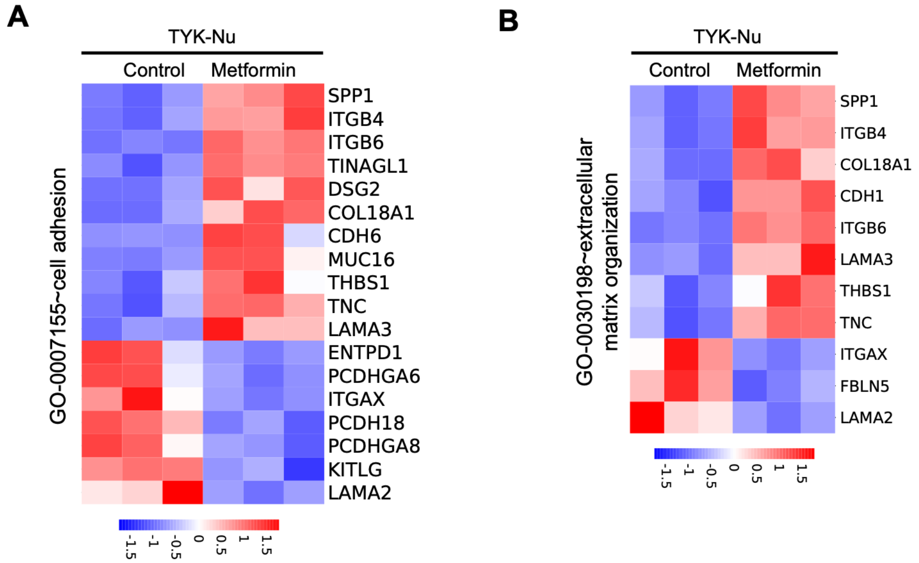

Figure S2. (A) Heatmap showing differentially regulated genes in metformin treated TYK-Nu cells related to cell adhesion. (B) Heatmap showing differentially regulated genes in metformin treated TYK-Nu cells related to extracellular matrix reorganization.

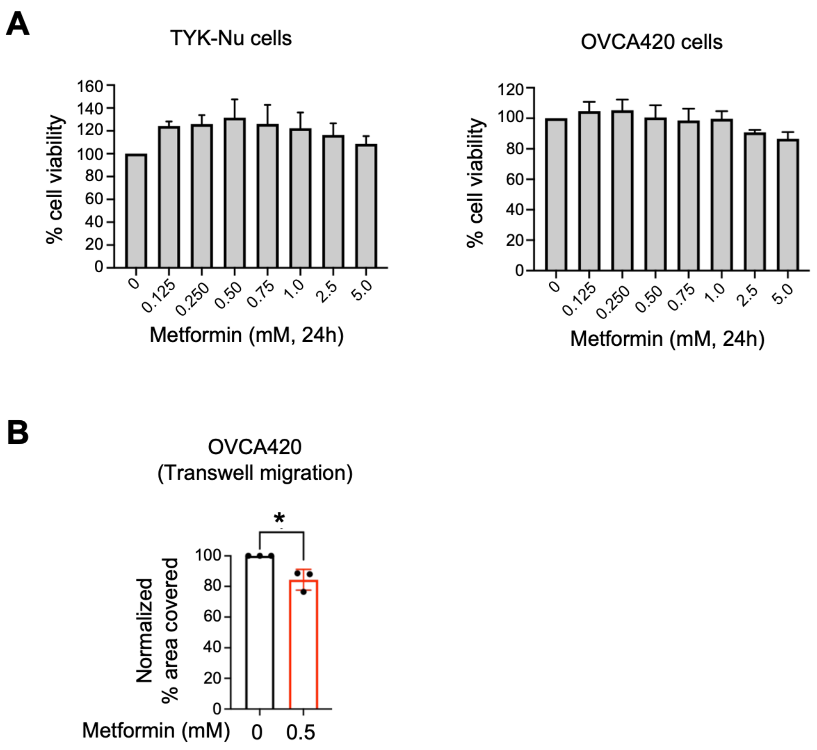

Figure S3. (A) WST-1 data shows that 24-hour metformin treatment does not alter cell viability of TYK-Nu and OVCA420 cells. (B) Quantified transwell migration data show that 0.5 mM metformin causes a decrease in OVCA420 cell migration.

Supplemental Table Legends

Table S1. Differentially expressed genes identified by RNA-seq analysis of OVCA420 cells following treatment with 5 mM metformin.

Table S2. Differentially expressed genes identified by RNA-seq analysis of TYK-Nu cells following treatment with 5 mM metformin.

Table S3. Complete list of GO terms enriched among differentially expressed genes identified by RNA-seq analysis of OVCA420 cells following treatment with 5 mM metformin.

Table S4. Complete list of GO terms enriched among differentially expressed genes identified by RNA-seq analysis of TYK-Nu cells following treatment with 5 mM metformin.
